# Multiple *FGF4* Retrocopies Recently Derived within Canids

**DOI:** 10.3390/genes11080839

**Published:** 2020-07-23

**Authors:** Kevin Batcher, Peter Dickinson, Kimberly Maciejczyk, Kristin Brzeski, Sheida Hadji Rasouliha, Anna Letko, Cord Drögemüller, Tosso Leeb, Danika Bannasch

**Affiliations:** 1 Department of Population Health and Reproduction, University of California-Davis, Davis, CA 95616, USA; klbatcher@ucdavis.edu (K.B.); kmaciejczyk@ucdavis.edu (K.M.); 2 Department of Surgical and Radiological Sciences, University of California-Davis, Davis, CA 95616, USA; pjdickinson@ucdavis.edu; 3 College of Forest Resources and Environmental Science, Michigan Technological University, Houghton, MI 49931, USA; kbrzeski@mtu.edu; 4 Institute of Genetics, Vetsuisse Faculty, University of Bern, 3012 Bern, Switzerland; sheida.hadjirasouliha44@gmail.com (S.H.R.); anna.letko@vetsuisse.unibe.ch (A.L.); cord.droegemueller@vetsuisse.unibe.ch (C.D.); tosso.leeb@vetsuisse.unibe.ch (T.L.)

**Keywords:** *Canis lupus* familiaris, *FGF4*, retrocopy, retrogene, pseudogene, retrotransposition, duplication

## Abstract

Two transcribed retrocopies of the fibroblast growth factor 4 (*FGF4*) gene have previously been described in the domestic dog. An *FGF4* retrocopy on chr18 is associated with disproportionate dwarfism, while an *FGF4* retrocopy on chr12 is associated with both disproportionate dwarfism and intervertebral disc disease (IVDD). In this study, whole-genome sequencing data were queried to identify other *FGF4* retrocopies that could be contributing to phenotypic diversity in canids. Additionally, dogs with surgically confirmed IVDD were assayed for novel *FGF4* retrocopies. Five additional and distinct *FGF4* retrocopies were identified in canids including a copy unique to red wolves (*Canis rufus*). The *FGF4* retrocopies identified in domestic dogs were identical to domestic dog *FGF4* haplotypes, which are distinct from modern wolf *FGF4* haplotypes, indicating that these retrotransposition events likely occurred after domestication. The identification of multiple, full length *FGF4* retrocopies with open reading frames in canids indicates that gene retrotransposition events occur much more frequently than previously thought and provide a mechanism for continued genetic and phenotypic diversity in canids.

## 1. Introduction

Gene retrocopies, often previously referred to as processed pseudogenes, are formed through the mRNA-mediated gene duplication of cellular gene transcripts [1]. In mammals, this process is mediated by long interspersed nuclear elements 1 (L1) acting in trans [2,3]. L1s are the only autonomous, retrotransposable elements still active today in mammals, and while over 100 active, full-length L1s have been identified in humans, dogs have more than 200 active L1s [4]. L1 insertion is accomplished through target primed reverse transcription, a process that results in duplication of genomic DNA at the insertion site, called a target site duplication (TSD) [5]. Because gene retrocopies are formed from processed mRNA, they also lack introns and contain a polyA tail, features that distinguish them from their parental gene. Although retrocopy insertions can occur anywhere in the genome, the L1 machinery shows a preference for the TTAAAA consensus sequence as an insertion site [3,6].

Retrocopies are more likely to come from highly expressed genes [7], with some genes having over a dozen retrocopies [8]. Most of the retrocopies present in any given reference genome arose millions of years ago and have since acquired numerous sequence variants differentiating them from their parent genes [8]. While many retrocopies have also lost their open reading frame (ORF), L1 is still actively producing retrocopies with intact ORFs in mammalian genomes. Hundreds of recent, polymorphic gene retrocopies have been reported in humans and mice [9,10,11]. Notably, polymorphic retrocopies were more common in mice than in humans, consistent with mice having more active L1s [9]. A recent survey of retrocopies in the canfam3 reference genome has identified over 3000 retrocopies, 476 of which were intact [12], and several gene retrocopies have also been identified on the canine Y chromosome [13]. However, it is still unclear how many recent, polymorphic retrocopies are in canids that are not present in the canfam3 reference genome.

Two expressed, polymorphic fibroblast growth factor 4 gene (*FGF4*) retrocopies have been described previously in dogs on chr18 [14] and chr12 [15], referred to as *FGF4*L1 (CFA18) and *FGF4*L2 (CFA12) in this study. Both *FGF4*L1 and *FGF4*L2 are associated with forms of disproportionate dwarfism that are common across many popular dog breeds, and there is evidence that these genes have been under selection owing their strong phenotypic effects [16,17]. *FGF4*L2 has also been associated with canine chondrodystrophy, a disorder characterized by premature degeneration of the intervertebral discs, which predisposes affected dogs to intervertebral disc herniation [18]. However, chondroid disc degeneration can also be seen in dogs without *FGF4*L2, indicating the possibility of alternative risk loci for the disorder [19].

Because two recent, functional *FGF4* retrocopies had already been described in dogs, we hypothesized that more *FGF4* retrocopies could be segregated across dog breeds, which may contribute to limb morphology and/or disc disease. Previous *FGF4* retrocopies were identified following genome-wide associations for disproportionate dwarfism. In the current study, two approaches were utilized to identify additional *FGF4* retrocopies in dogs. First, discordant read mapping of paired-end Illumina reads from publicly available whole-genome sequence data was used to identify additional polymorphic *FGF4* retrocopies in canid genomes that would not be identified by common variant calling techniques. The second approach was to perform exon to exon polymerase chain reaction (PCR) to identify the presence of an intron-less retrocopy, followed by inverse PCR to identify the site of insertion. Five additional *FGF4* retrocopies were then identified, sequenced, and characterized.

## 2. Materials and Methods

### 2.1. FGF4 Retrocopy Discovery in Whole-Genome Sequence Data

Data from six BioProjects (PRJNA448733, PRJEB16012, PRJNA288568, PRJNA377155, PRJEB20635, and PRJEB32865) were utilized for this approach [20,21,22,23,24,25]. This included 1125 individuals from 160 different breeds, as well as 101 indigenous dogs, 141 wolves, and 3 coyotes (Supplemental Table S1). The canine reference genome, CanFam3, does not contain any full length *FGF4* retrocopies, and thus all reads coming from *FGF4* retrocopies are aligned to the parental *FGF4* gene locus. To identify any such novel *FGF4* retrocopies, aligned paired end Illumina sequence data in the region surrounding the *FGF4* gene (CanFam3 chr18:48,412,000–48,418,000) were downloaded from the Sequence Read Archive and analyzed. Sequencing files were viewed in Integrative Genomics Viewer [26]. Discordant paired end reads mapping from exon to exon (Supplemental Figure S1 shown in red) are indicative of the presence of an *FGF4* gene retrocopy somewhere in the genome as retrocopies lack introns, while discordant paired end reads, wherein one mate maps to the *FGF4* gene locus and the other mate maps to another region of the genome, are indicative of the putative insertion site for an *FGF4* retrocopy (Supplemental Figure S1 shown in teal). The presence of both forms of discordant reads was used as an indication of an *FGF4* retrocopy insertion.

### 2.2. FGF4 Retrocopy Discovery in Clinical Cases

Whole-genome sequence data were not available for any of the individuals treated by surgical decompression for presumed IVDD. Therefore, a molecular approach was developed to test for novel *FGF4* retrocopies in DNA samples. A total of 164 surgical cases that were previously shown to have 0 copies of *FGF4*L1 and *FGF4*L2 were used for novel *FGF4* retrocopy discovery [19]. The presence of *FGF4* retrocopies was tested by amplifying the region between exon 1 and exon 3 of *FGF4* (Supplemental Table S2). The identification of a reduced size, intron-less product indicates the presence of an *FGF4* retrocopy (Supplemental Figure S2). When an individual tested positive for an *FGF4* retrocopy and negative for the two known *FGF4* retrocopy insertions, inverse PCR [27] was then performed to identify the insertion site of the *FGF4* retrocopy. For inverse PCR, 1 μg of genomic DNA was digested with the MboI restriction enzyme according to the manufacturer’s instructions (New England Biolabs, Ipswich, MA, USA), and fragments were then circularized by ligation at final concentrations varying between 1 and 10 ng/μL using T4 DNA ligase according to the manufacturer’s instructions for sticky end ligation (New England Biolabs, Ipswich, MA, USA). A set of inverted primers were designed that amplified circular DNA fragments containing the 5′ end of the *FGF4* retrocopy insertion (Supplemental Table S2). PCR was then performed using LongAmp Taq DNA polymerase according to the manufacturer’s instructions (New England Biolabs, Ipswich, MA, USA). PCR products were visualized by gel electrophoresis and isolated for Sanger sequencing using a QIAquick Gel Extraction Kit (Qiagen, Valencia, CA, USA). All PCR primers were designed using primer3 (http://bioinfo.ut.ee/primer3/) [28].

### 2.3. Sequencing and Comparitive Analysis of FGF4 Retrocopies

All canine DNA samples used for retrocopy sequencing and subsequent population genotyping of the *FGF4* retrocopies came from the Bannasch Canine Repository and were obtained under UC Davis Animal Care and Use Committee protocol 18,561 [19] (Supplemental Table S3). Red wolf tissue samples for DNA extraction were obtained with the approval of the United States Fish and Wildlife Services. PCR primers were designed to flank the insertion sites of *FGF4* retrocopies identified via discordant paired end reads or inverse PCR (Supplemental Table S1). Entire retrocopy insertions were then amplified through PCR using LongAmp Taq DNA polymerase according to the manufacturer’s instructions (New England Biolabs, Ipswich, MA, USA). The full sequence of each retrocopy was obtained through Sanger sequencing using a series of internal primers (Supplemental Table S2). Variants in the parental *FGF4* gene were observed in a dataset of 722 canids to determine which single-nucleotide variants (SNVs) were unique to *FGF4* retrocopies [20].

### 2.4. Conservation at Insertion Sites

Evolutionarily conserved elements (ECR) near the *FGF4* retrocopy insertion sites were defined using the 4-Way Multiz Alignment & Conservation track for CanFam2 on the UCSC genome browser [29], which shows a measure of evolutionary conservation between dog, human, mouse, and rat genomes using Multiz alignment [30].

### 2.5. Population Genotyping

Breeds were selected for population genotyping based on the breeds in which they were identified, excluding breeds where whole-genome sequencing data indicated they did not contain any FGF4 retrocopies. PCR assays utilizing three primers per assay were designed for each *FGF4* retrocopy for population genotyping, as previously described [15]. In each assay, a shared internal primer at the 3′ end of the *FGF4* retrocopy produces a different size amplicon when the retrocopy is present (Supplemental Table S1).

### 2.6. Height Measurements

Height was measured in selected cases to determine if *FGF4* retrocopies had any effect on height. All height measurements were performed by the same individual using a standard wicket (height measuring device for dogs). Multivariable linear regression was performed in R studio using the generalized linear model function with sex and *FGF4* genotype included to identify any association with height.

## 3. Results

### 3.1. FGF4 Retrocopy Discovery from Whole-Genome Sequence Data

In addition to the two known *FGF4* retrocopies, *FGF4*L1 and *FGF4*L2, evidence for four additional *FGF4* retrocopies in canids was observed in the whole-genome sequence dataset (Table 1). The novel *FGF4* retrocopies include a copy on CFA27 (*FGF4*L3) seen in three Nova Scotia Duck Tolling Retrievers (NSDTR); a copy on CFA22 (*FGF4*L4) seen in two Norwich Terriers; a copy on CFA13 (*FGF4*L5) seen in a Belgian Malinois and a Dutch Shepherd; and a copy on CFA36 (*FGF4*L6) seen in two red wolves. Sequence read archive (SRA) accession numbers for these individuals are available in Supplemental Table S4.

Discordant reads were also observed at the 3′ end of the *FGF4* gene locus aligning to a partial *FGF4* retrocopy insertion in the CanFam3 reference genome at chr7:68,372,263–68,373,442. To confirm whether this was a real *FGF4* retrocopy fragment or a mistake in the reference assembly, primers were designed flanking the insertion and the region was amplified in six Boxers. Five were heterozygous for the CFA7 partial *FGF4* retrocopy insertion, and Sanger sequencing confirmed the sequence matched the reference genome. Because this retrocopy only contains the 3′ UTR of the gene and has no ORF, this retrocopy fragment was not considered for further analysis.

### 3.2. FGF4 Retrocopy Discovery in Dogs Treated for Disc Disease

A surgically treated population of 164 individuals that had neither *FGF4*L1 nor *FGF4*L2 was then tested for the presence of any *FGF4* retrocopy using an exon–exon PCR assay. Four of these individuals tested positive for the presence of an *FGF4* retrocopy. These samples were first tested for the other newly discovered *FGF4* retrocopies. One sample, a Shetland Sheepdog, was heterozygous for *FGF4*L5. The medical history of this individual indicates that it received a hemilaminectomy to treat a mass that was not disc-related.

The three remaining dogs were all Pit Bull Terrier mixes that had received hemilaminectomies for IVDD at relatively young ages (age at time of surgery of 3, 5, and 8 years), and none of the newly discovered or previously defined *FGF4* retrocopies were present in these individuals, indicating they contained a novel *FGF4* retrocopy. Inverse PCR was then performed to discover the insertion site of the novel *FGF4* retrocopies in these individuals, which was on CFA13 (*FGF4*L7) at approximately CFA13:25,020,600. The three dogs were all heterozygous for *FGF4*L7, and Sanger sequencing revealed that *FGF4*L7 is a full length *FGF4* retrocopy.

### 3.3. Comparative Analysis of FGF4 Retrocopies

Novel *FGF4* retrocopies were confirmed through PCR amplification and sequencing. The genomic location for the *FGF4* retrocopies, their TSD, and genomic sequence surrounding the TSD are shown in Table 1. Exact TSD length varied from 11 to 17 bases, with a median of 15 bp. The loosely conserved L1 consensus insertion site sequence (TTAAAA) was only observed at the *FGF4L5* insertion site. Insertion sites for 6/7 of the *FGF4* retrocopies had a low G/C content compared with the Canfam3 average of 41.3% (Table 1). All *FGF4* retrocopies inserted into intergenic regions of the genome. Both *FGF4L1* and *FGF4L3* inserted into a LINE element, while *FGF4L4* inserted into a long terminal repeat (LTR). The number of evolutionarily conserved regions within 2.5 kb of the insertion sites is also reported in Table 1.

**Figure 1:**
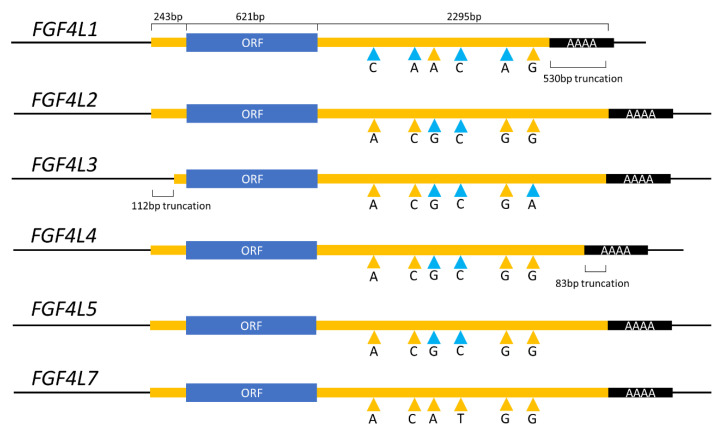
Comparison of the six full length *FGF4* retrocopies identified in domestic dogs. From left to right, the letters with colored arrowheads represent variants within the 3′ UTR of the *FGF4* gene at genomic locations CFA18:48,415,400A>C; CFA18:48,415,405C>A; CFA18:48,415,585A>G; CFA18:48,415,608T>C; CFA18:48,415,661G>A; and CFA18:48,416,537G>A. SNV colored in blue represent non-reference alleles. ORF, open reading frame.

Comparison of each *FGF4* retrocopy to the parental *FGF4* sequence showed that each novel copy has a fully conserved ORF (Figure 1). The 5′ UTR of *FGF4*L3 is truncated by 112 bp compared with the other retrocopies, and the 3′ UTR in both *FGF4*L1 and *FGF4*L4 is truncated by 530 bp and 83 bp. No single-nucleotide variants (SNVs) were identified in either the ORF or the 5′ UTR of any of the retrocopies. However, six SNVs were identified in the 3′ UTR that differed from the reference genome *FGF4* gene sequence. Analysis of a whole-genome sequencing variant calling dataset from 722 canids indicated that these SNVs are also present at the parental *FGF4* gene (Supplemental Table S5). Therefore, no SNV specific to any of the dog *FGF4* retrocopies was identified. Rather, the differences between *FGF4* retrocopies are owing to different haplotypes of the parental *FGF4* gene from which the retrocopies formed. Notably, the 3′ end of the parental *FGF4* gene in wolves contains several SNV not identified in any domestic dogs (Supplemental Table S5).

The red wolf *FGF4*L6 3′ UTR sequence contained two single nucleotide indels not observed in any domestic dog *FGF4* sequences: a deletion (CFA18:48,415,685delA) and an insertion (CFA18:48,416,575_48,416,576insA). These indels were not identified in any canids other than the two red wolves in a whole-genome sequencing variant calling dataset, which included 46 gray wolves. The parental *FGF4* locus was sequenced in seven red wolves to determine whether these variants also exist in the parental gene in red wolves or if they are unique to the retrocopy. While three individuals were heterozygous for the CFA18:48,416,575T>TA insertion at the parental *FGF4* gene, CFA18:48,415,685CA C was not identified in any of the parental *FGF4* sequences, indicating this variant may have occurred after retrotransposition and may be unique to *FGF4*L6.

### 3.4. Population Genotyping of Novel FGF4 Retrocopies

A targeted population genotyping approach based on the breeds in which *FGF4* retrocopies were identified was utilized to determine allele frequencies of the *FGF4* retrocopies. A complete list of *FGF4* retrocopy genotyping results is available in Supplemental Table S3. *FGF4*L3 was only observed in the NSDTR breed in the whole-genome sequencing dataset, and was thus tested in 100 randomly selected NSDTR. The allele frequency of *FGF4*L3 was 8.5% in the NSDTR.

*FGF4*L4 had an allele frequency of 16.7% in Norwich Terriers (*n* = 30). Further testing for *FGF4*L4 in related terrier breeds identified this retrocopy in Norfolk Terriers (*n* = 10, allele frequency 30%), Border Terriers (*n* = 32, allele frequency 71.9%), and Skye Terriers (*n* = 10, allele frequency 5%). Given the previous association of *FGF4* retrogenes with skeletal dysplasia, *FGF4*L4 genotype was also compared to height at the withers in 24 Border Terriers using multiple linear regression. The regression analysis identified no significant association between *FGF4*L4 and height in Border Terriers (*p* = 0.877, *n* = 24), although only one homozygous wild type individual was included (Supplemental Figure S3).

*FGF4L5* was not identified in any other Shetland Sheepdogs (*n* = 58) or Belgian Malinois (*n* = 14). Australian Shepherds (*n* = 19) and Anatolian Shepherd dogs (*n* = 5) also tested negative for *FGF4L5*. No additional Dutch Shepherd samples were available for population genotyping of *FGF4L5* in the breed. *FGF4L6* was tested in 14 red wolf samples, 5 of which were heterozygous (allele frequency 15.6%).

Pit Bull Terriers and Pit Terrier Mixes were then tested for *FGF4L7* (*n* = 201), and all tested negative for the retrocopy. Because *FGF4L7* was identified in dogs treated for IVDD and could be contributing to the disorder, all mixed breed dogs from the Bannasch Canine Repository that had been treated surgically for IVDD were also tested for *FGF4L7* (*n* = 55), all of which tested negative. However, two discordant reads mapping to the *FGF4L7* were subsequently identified in the whole-genome sequence data of a single Chinese village dog (SRR7107669). Several breeds developed in Asia were then tested for *FGF4L7*, including Chow Chow (*n* = 22), Pugs (*n* = 9), Pekingese (*n* = 8), and Tibetan terriers (*n* = 6), none of which tested positive. However, *FGF4*L7 was identified in Chinese Shar-Pei (*n* = 22, allele frequency 34.1%).

## 4. Discussion

Multiple recently transposed *FGF4* retrocopies exist in canids in addition to the previously identified *FGF4*L1 and *FGF4*L2. Novel retrocopies appear to be breed or breed group specific, contain intact ORFs, and have not accrued mutations that differentiate them from parental *FGF4* gene haplotypes. The *FGF4* retrocopies were retrotransposed from *FGF4* genes with distinct haplotypes, indicating that the same copy has not been retrotransposed multiple times. It is unclear whether any of these novel copies are expressed retrogenes, or in what tissue types they could be expressed. *FGF4*L7 was identified in three dogs treated surgically for IVDD, however, the significance relative to IVDD is unknown. The majority of IVDD surgical cases examined in this study that were not explained by *FGF4*L2 were found to have no *FGF4* retrocopies, indicating that there are risk factors other than *FGF4* retrocopies that predispose dogs to IVDD.

Evidence for the expression of both *FGF4*L1 and *FGF4*L2 has indicated that the *FGF4* retrocopies are capable of expression [14,15]. The 5′ end of the *FGF4* gene is GC-rich and contains many evolutionarily conserved transcription factor binding sites that were previously hypothesized to be conducive towards expression of the retrocopies [15]. Thus, the 5′ end truncation of the *FGF4*L3 retrocopy likely affects expression. It has also been reported that the expression of retrocopies is highly dependent on the genomic environment of the insertion sites [31]. Both *FGF4*L1 and *FGF4*L2 have inserted into regions containing nearby evolutionarily conserved elements (ECRs). Similarly, ECRs at all but one of the *FGF4* retrocopy insertion sites may be conducive towards expression. The different genomic context at the insertion sites for *FGF4*L1 and *FGF4*L2 could also explain the different phenotypes between the copies. If expressed, the novel *FGF4* retrocopies may show unique expression profiles, resulting in phenotypic associations other than height and IVDD. *FGF4* is involved in several cellular processes including cell growth, tissue repair, tumor growth and invasion, and is also a well-known proto-oncogene [32,33].

Although *FGF4*L2 has been shown to have a major association with IVDD [15,19,34], clinically significant IVDD has been reported in dogs lacking the *FGF4*L2 retrogene, implicating alternate causative factors [19]. Additional *FGF4* retrogenes are logical candidates for these *FGF4*L2 negative IVDD cases, however the additional *FGF4* retrocopies identified in this study do not appear to provide a compelling explanation for this group of dogs owing to the limited frequency of the retrogenes in affected animals. Although *FGF4*L7 was identified in three dogs treated surgically for IVDD, it was not seen in any other breeds in the surgically treated data set, and the breed with the highest identified allele frequency (Shar-Pei; 0.341) is not known to be among the breeds highly predisposed to IVDD [35]. Similarly, clinical IVDD is uncommon in Border Terriers and Norfolk Terriers, which had the highest allele frequency of the *FGF4*L4 retrogene. Interestingly, *FGF4*L7 inserted 5 Mb downstream from the HAS2 gene, a gene that has been implicated in the Shar-Pei wrinkled skin phenotype as well as Familial Shar-Pei Fever [36]. Strong selection in this region of the genome in Shar Peis could explain the high allele frequency of *FGF4*L7 in the breed.

While *FGF4*L4 was not found to be associated with height in Border Terriers, the majority of Border Terriers tested had either one or two copies of *FGF4*L4, and only one individual with 0 copies was identified. If the retrocopy has a dominant effect on height in the breed, more homozygous wild type individuals will need to be measured to determine any effect. *FGF4*L4 was also found at low allele frequencies in other related terrier breeds, including the Skye, Norwich, and Norfolk Terriers, and may have originated in a common progenitor to the terrier breed group. As dog breeds are known to be highly inbred [37,38], a high allele frequency alone does not indicate selection, as it could be the result of random genetic drift followed by decreasing genetic diversity, as characterizes purebred dogs. Interestingly, *FGF4*L1 is also very common in Norwich and Norfolk Terriers [14], and the Skye Terriers used in this study were homozygous for both *FGF4*L1 and *FGF4*L2, making them the first breed to be identified with three *FGF4* retrocopies.

All the *FGF4* retrocopies in canids appear to have been very recently retrotransposed with no new mutations differentiating them from the parental *FGF4* gene. Even the red wolf *FGF4* retrocopy, *FGF4*L6, is nearly identical to the red wolf specific *FGF4* haplotype. Dating the *FGF4* retrocopy insertions is difficult owing to their short length (3.2 kbp) and sequence identity to the parental gene sequence; however, the *FGF4* retrocopies are identical to canine-specific *FGF4* gene haplotypes, which are distinct from modern wolf *FGF4* haplotypes (Supplemental Table S5). This could indicate that the dog retrocopies occurred after domestication. Recently inserted, fully intact retrocopies such as the *FGF4* retrocopies are very uncommon in reference genomes. Studies have found that less than 18% of the retrocopies in the human reference have a fully intact ORF, while only 1% of retrocopies share greater than 99% of their DNA sequence with their parental gene [31,39]. However, these studies have focused on analyzing reference genomes, which miss polymorphic retrocopies that are more likely to be recent, such as the *FGF4* retrocopies in canids, which are not found in CanFam3. It is possible that some unique aspects of the *FGF4* gene increase its rate of L1 mediated retrotransposition. A search for *FGF4* through a database of all retrocopies identified in over 40 mammalian reference genomes reveals that a squirrel (*Ictidomys tridecemlineatus*) and a hedgehog (*Echinops telfairi*) also have *FGF4* retrocopies, although they are only 61.2% and 90.6% identical to the parental genes, indicating they are not recent [8], but it is unknown whether other species have polymorphic *FGF4* retrocopies not found in their reference genomes. Another possibility is that L1 mediated gene retrotransposition in general is occurring more frequently in canids. If this was the case, recent, polymorphic retrocopies may be more common in canids in a greater number of genes than just *FGF4*.

While next generation sequencing allows for the detection of polymorphic gene retrocopies, they often go unidentified or misidentified by common variant calling methods [40]. However, more careful analysis of discordant Illumina paired-end reads has shown they are more common than previously thought [41,42]. As with *FGF4*L1 and *FGF4*L2, retrocopies of other genes may have phenotypic consequences. As such, the possibility of retrocopy insertions should be considered when scanning critical intervals for disease trait associations. Recently inserted gene retrocopies can result in overexpression of the parental gene product, resulting in gain of function, which could be deleterious [43]. In this study, whole-genome sequence data were successfully used to identify novel, polymorphic retrocopies of the *FGF4* gene; a similar approach could be generalized to all genes to identify other polymorphic gene retrocopies in canids. Similar to the *FGF4* retrocopies, other polymorphic retrocopies may play an important role in both breed health and phenotypic variation across dogs.

## Figures and Tables

**Table 1:** Genomic sequence at fibroblast growth factor 4 gene (*FGF4*) retrocopy insertion sites in canids. Target site duplications (TSDs) are in bold and underlined. Ten bases upstream and downstream from the TSD are included, as well as the strand orientation of the retrocopy, G/C content of the region, and evolutionarily conserved elements (ECR) within 2.5 kb of the insertion site. *FGF4* retrocopies were identified by GWAS, discordant read mapping (DRM), and inverse PCR.

Name	Location	Sequence at Insertion Site	Strand	G/C	ECR	Method
***FGF4L1***	Chr18:20,443,703–20,443,735	ACCATGAAAT**AAGTCAGACAGAG**AAAGACAAGT	+	36.4	2	GWAS [14]
***FGF4L2***	Chr12:33,710,158–33,710,188	ATTCCTATTC**AAGTGCTTTGA**ACTCTTCAAA	+	32.3	1	GWAS [15]
***FGF4L3***	Chr27:24,834,102–24,834,135	TGAGAATACT**CAGGGACCATTTCT**ATTGACTTTT	-	35.3	0	DRM
***FGF4L4***	Chr22:47,761,852–47,761,888	TGTCTTTGTC**AAGAATATTCTGGTTGT**GAGTAATAGA	+	32.4	2	DRM
***FGF4L5***	Chr13:28,020,009–28,020,044	GCAGTTTCTT**AAAACTTAGAGGAACA**AAGTAGCTTG	+	36.1	6	DRM
***FGF4L6***	Chr36:11,456,175–11,456,208	AAAGCATTAA**TTACCAAAGTACTA**TTTCATAACT	+	23.5	1	DRM
***FGF4L7***	Chr13:25,020,597–25,020,632	GAATCGTGTT**TAAGAAGGGGTGGTAT**GACTTGCCCT	-	47.2	3	Inverse PCR

## Supplementary Materials

The following are available online at https://www.mdpi.com/2073-4425/11/8/839/s1, Figure S1: Retrocopy discovery from whole-genome sequencing data; Figure S2: *FGF4* genotyping assay; Figure S3: Height in Border Terriers; Table S1: Breed list of whole-genome sequence files analyzed; Table S2: *FGF4* retrocopy primers; Table S3: Breed list and *FGF4* retrocopy genotype results; Table S4: SRA accession numbers for canids with novel *FGF4* retrocopies; Table S5: SNV at the *FGF4* gene locus in 722 canids.
