# Supplementary figures and images for: Multiple FGF4 Retrocopies Recently Derived within Canids
Source: Genes (Basel). 2020 Jul 23;11(8):839. doi: 10.3390/genes11080839 (PMC7465015; doi:10.3390/genes11080839)

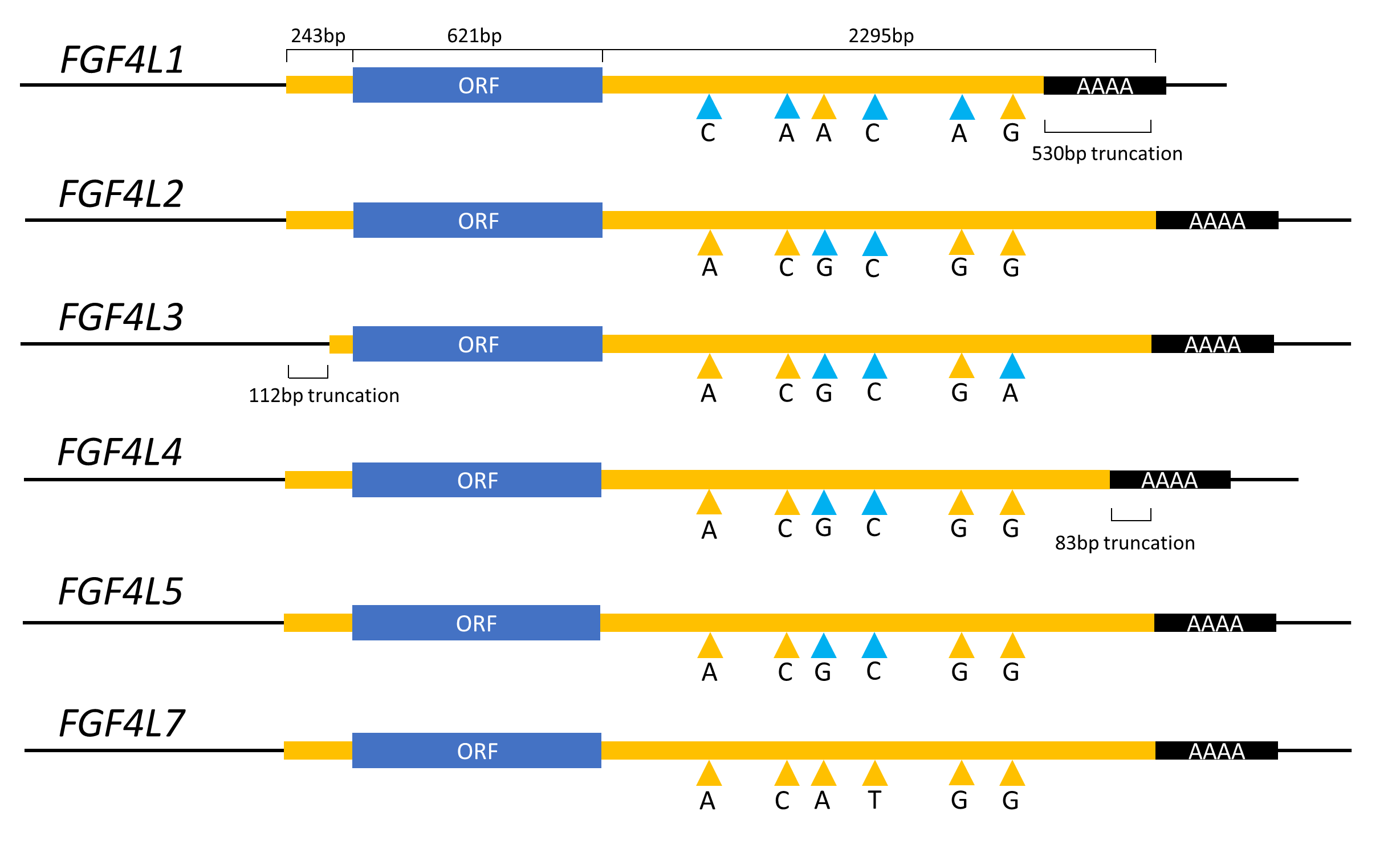

Supplement: Supplementary file 1 [file genes-11-00839-s001.zip › Figure_and_tables/figure_1.tif]

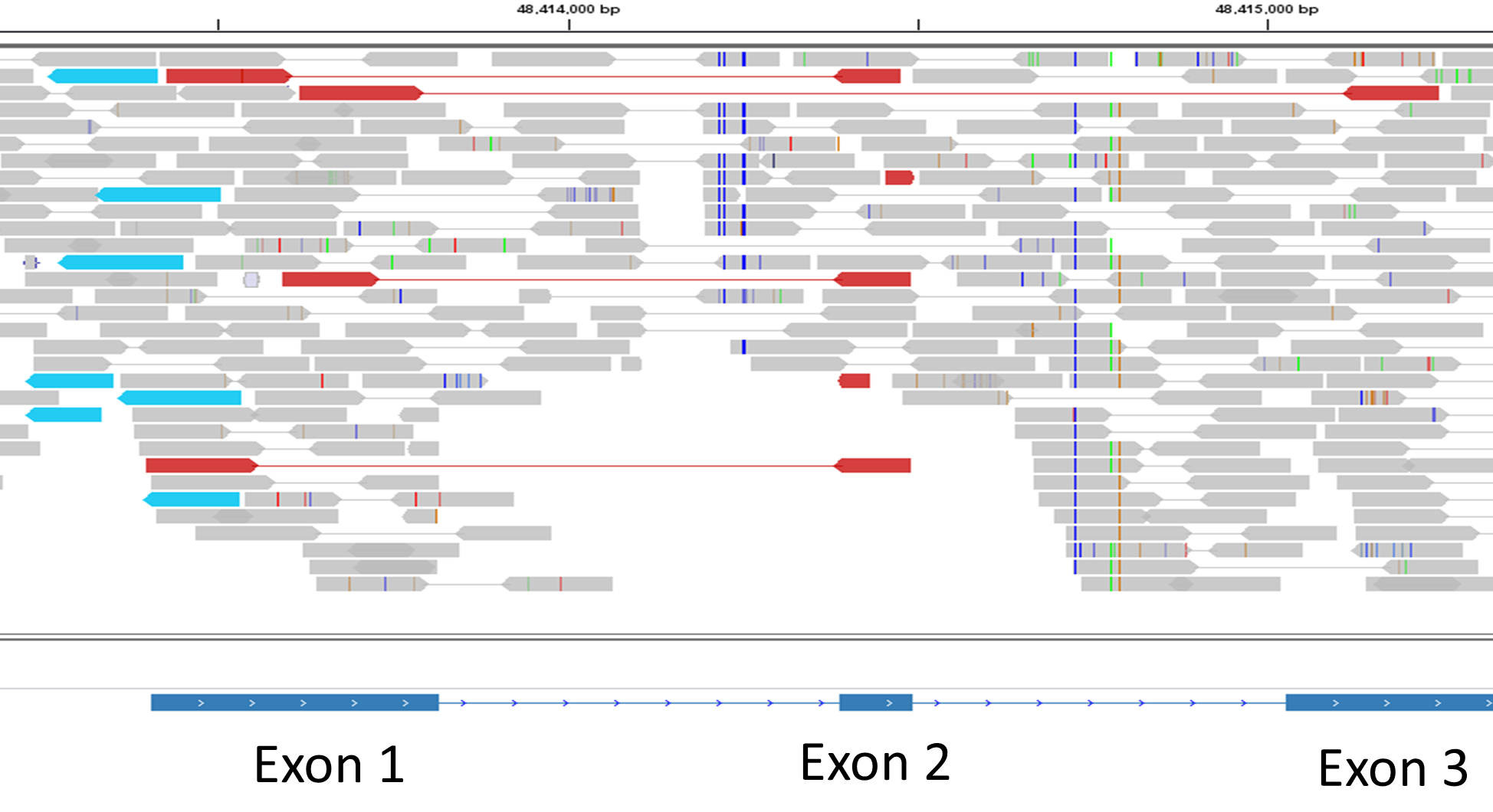

Supplement: Supplementary file 1 [file genes-11-00839-s001.zip › Figure_and_tables/S1_fig.tif]

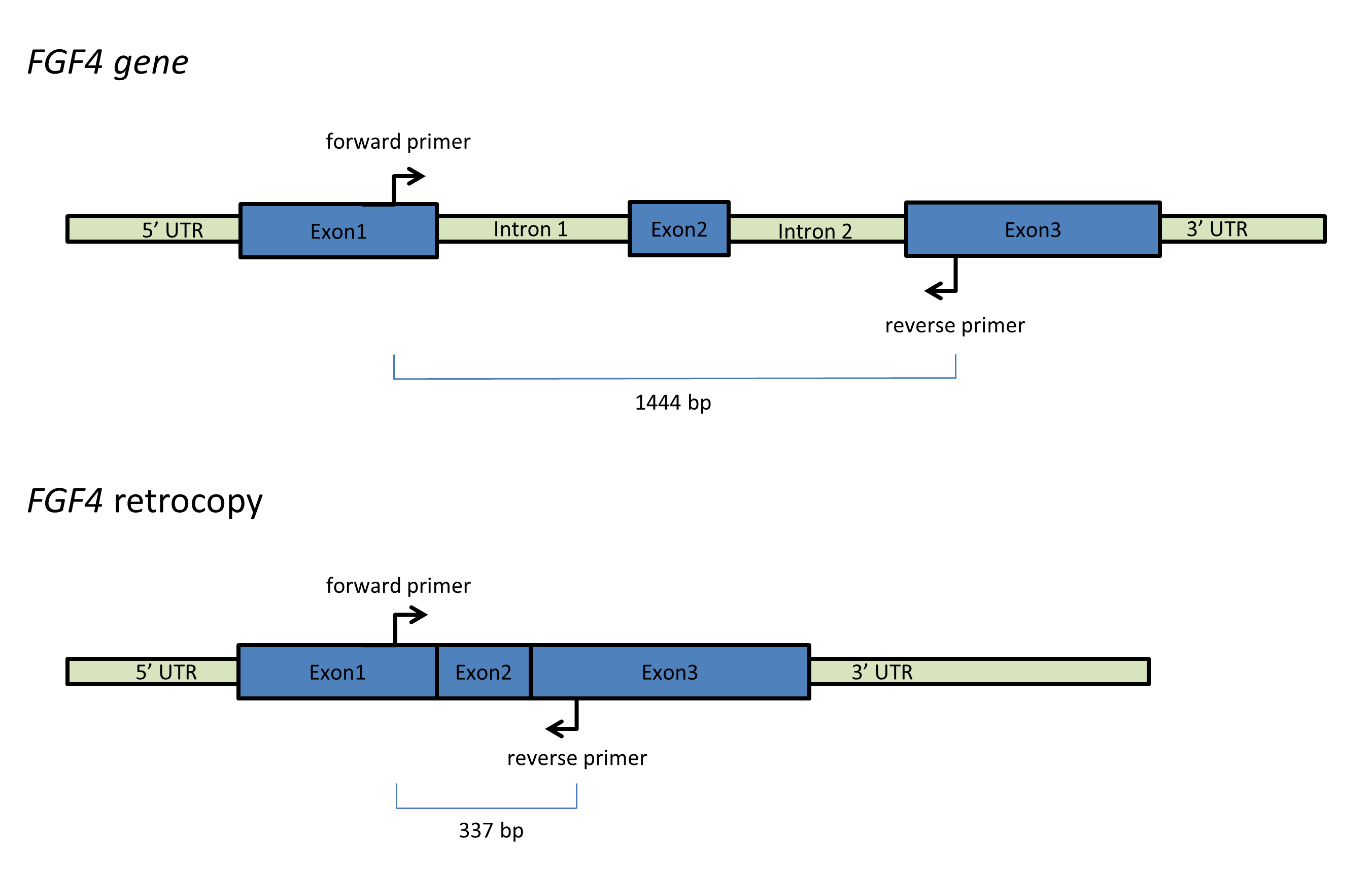

Supplement: Supplementary file 1 [file genes-11-00839-s001.zip › Figure_and_tables/S2_fig.tif]

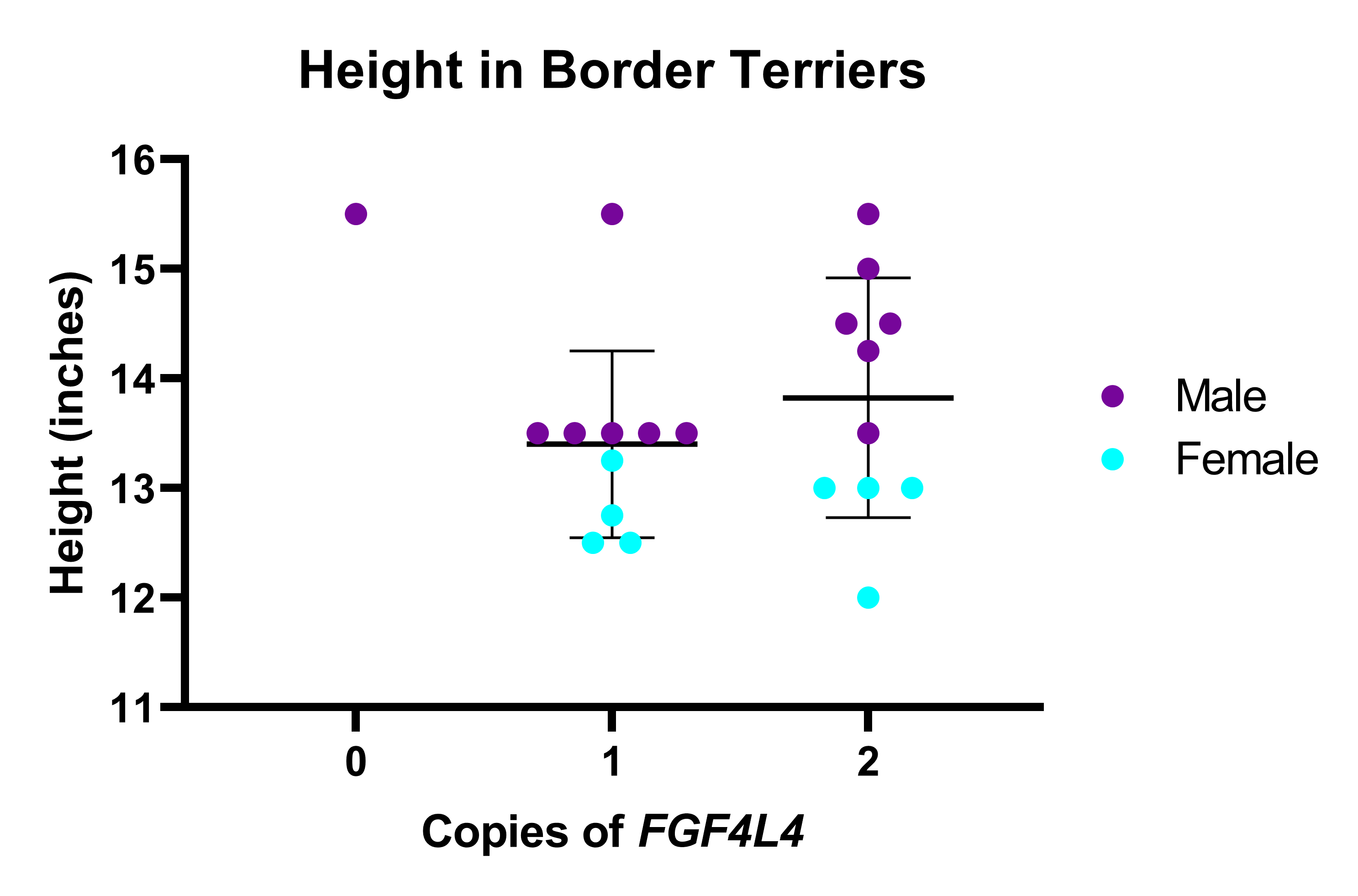

Supplement: Supplementary file 1 [file genes-11-00839-s001.zip › Figure_and_tables/S3_fig.tif]
